# The Application of High-Hydrostatic-Pressure Processing to Improve the Quality of Baked Products: A Review

**DOI:** 10.3390/foods13010130

**Published:** 2023-12-29

**Authors:** Ángel L. Gutiérrez, Daniel Rico, Felicidad Ronda, Pedro A. Caballero, Ana Belén Martín-Diana

**Affiliations:** 1 Food Technology, Department of Agriculture and Forestry Engineering, University of Valladolid, 34004 Palencia, Spain; angelluis.gutierrez@uva.es (Á.L.G.); mfronda@uva.es (F.R.); 2 Agrarian Technological Institute of Castilla and Leon (ITACyL), Ctra. Burgos Km 119, Finca Zamadueñas, 47071 Valladolid, Spain; ricbarda@itacyl.es (D.R.); mardiaan@itacyl.es (A.B.M.-D.)

**Keywords:** high hydrostatic pressure, plant-based ingredients, physical modifications, functional and nutritional properties, baked products

## Abstract

The current trend in the food industry is towards “clean label” products with high sensory and nutritional quality. However, the inclusion of nutrient-rich ingredients in recipes often leads to sensory deficiencies in baked goods. To meet these requirements, physically modified flours are receiving more and more attention from bakery product developers. There are various findings in the literature on high hydrostatic pressure (HHP) technology, which can be used to modify various matrices so that they can be used as ingredients in the baking industry. HHP treatments can change the functionality of starches and proteins due to cold gelatinization and protein unfolding. As a result, the resulting ingredients are more suitable for nutrient-rich bakery formulations. This review describes the information available in the literature on HHP treatment conditions for ingredients used in the production of bakery products and analyses the changes in the techno-functional properties of these matrices, in particular their ability to act as structuring agents. The impact of HHP-treated ingredients on the quality of dough and bakery products and the effects on some nutritional properties of the treated matrices have been also analysed. The findings presented in this paper could be of particular interest to the bakery industry as they could be very useful in promoting the industrial application of HHP technology.

## 1. Introduction

Baking is one of the world’s most popular processing methods for starchy staples because it imparts specific sensory characteristics to the final product, which are widely accepted by consumers. Among these, flavour, aroma and texture are the most important and characteristic. Wheat flour-based bakery products are obtained from doughs, which, due to their unique mechanical properties in terms of viscoelasticity, cohesiveness and extensibility, offer particular machinability and gas retention capacity during fermentation, which is key to the development of products such as leavened bread. The functionality of wheat dough is mainly dependent on the proportion of the gluten-forming proteins glutenins and gliadins and their interactions with other flour components [1,2]. The use of refined or white wheat flour in breadmaking is common because it results in breads that are more appreciated by consumers for their sensory properties [3]. Similarly, commercial gluten-free bakery products are also based on starches and flours, mainly maize and white rice, respectively [4].

Refined flours, on the other hand, are nutritionally poorer than their whole counterparts because the milling process removes the germ and the outer seed coat or bran, which contain valuable nutritional elements such as proteins, dietary fibre, fat, micronutrients and bioactive compounds [5,6]. Fortified gluten and gluten-free bakery products that meet health-conscious consumers’ preferences are gaining a prominent place in the bakery market. It is widely recognised that fortified baked goods with nutrient-dense whole flours could be an effective strategy to meet the dietary requirements for fibre and other micronutrients generally limited in Westernised and celiac diets [3,5,7,8]. Sources of fortification can also be derived from minor cereals [9], pseudocereals [10], legumes [11,12] or other plant sources such as hemp [13]. Furthermore, the use of uncommon crops for this purpose could be an interesting alternative for farmers due to the potential added value of these crops. Increasing agricultural diversity could be a step towards a healthier ecosystem, reduced agricultural economic volatility [14] and more sustainable food chains [11], thus contributing to improving system productivity and sustainability [15].

However, this nutrient-enriching formulation strategy often results in lower sensory quality of the resulting breads [3]. Components such as phytic acid [3], phenolic compounds [6] or dietary fibres [2] impair proper flour functionality and dough yield. Fibres are particularly detrimental, as they can act as water competitors, hampering the necessary dough hydration. This effect produces a negative effect on dough rheology, reducing the elastic properties and inducing a more viscous behaviour, which leads to weaker doughs [5]. It also alters the water availability for starch gelatinisation, limiting the granule starch swelling and amylose leaching, which affects the formation of a proper crumb structure [16]. In addition, the presence of phenolic acids in the fibres, such as ferulic acid, could alter the functionality of the gluten network by increasing the extensibility of the dough. [5]. The final product often shows undesirable sensory properties such as reduced brightness, lower specific volume and increased crumb hardness [2]. Dosage level, particle size and botanical origin are conditions to be taken into account for the addition of fibre in baked product formulations [17]. In gluten-free (GF) bakery formulations, some adverse effects of the interaction of fibre with the gluten substitute used, usually hydrocolloids, should also be taken into account [18].

Studies aimed at improving the quality of fortified bread have investigated various alternatives. One of them is the addition of improvers, such as vital gluten, in combination with surfactants with or without shortening [19], a strategy that was not completely effective in counteracting loaf volume reduction and crumb grain impairment. The inclusion of natural materials, such as rosehip and cephalaria, has also been studied, with positive results in improving the rheological properties of whole wheat dough [20]. Some positive results were also observed with the addition of soluble dietary fibres (SDF) compared to insoluble ones (IDF)*,* such as longer dough development time [17] or higher volumes in gluten [21] and gluten-free bread [18].

In recent years, an increasing number of studies on the application of non-thermal emerging technologies (e.g., ultrasound, non-thermal plasma, ozonation, ultraviolet light, pulsed light or high hydrostatic pressure) to improve the quality of food products of plant origin have been reported [22,23,24]. In cereal-based matrices, these technologies can alter the main components of flour, protein and starch, enabling the production of physically modified ingredients [25,26]. The functionality of the plant material resulting from these processing methods is drawing attention to the production of baked goods. These ingredients could be a promising alternative to chemical additives and thus help reduce or even eliminate the use of preservatives and other synthetic additives, facilitating the industry’s goal of offering “clean label” products that are in high demand by conscious consumers looking for healthier diets and lifestyles [27].

High hydrostatic pressure (HHP), also known as cold pasteurisation, has been reported to exert significant effects on starch and protein polymers [27,28,29]. This technology offers interesting benefits in the food industry, not only in terms of shelf life extension but also in terms of preserving the natural flavour and nutrient profile of the original food material [30]. In addition, compared to thermal treatments, this technology uses less energy, which means less environmental impact [31]. HHP technology has also been proposed to improve textural properties, increase the bioavailability and bioaccessibility of bioactive compounds and minerals and reduce the risk of allergies in some food products [32].

There is now a growing body of research on HHP technology applied to cereal-derived matrices such as starch, flour or grains to physically modify their components and improve their native functionality [33,34,35,36,37]. In this review, a brief introduction to HHP technology is presented, followed by the effect of HHP application on the main flour constituents, starch and proteins. The aim of this review was also to address changes reported in the literature on the techno-functional properties of HHP-modified biopolymers, as well as on more complex matrices that could be used as bakery ingredients of high nutritional interest. The impact of using HHP-modified ingredients on the dough and the resulting breads was also deeply analysed. Finally, some strategies to improve the value of the nutritional profile in relation to the content of bioactive compounds of starch-rich food ingredients through HHP technology were also addressed.

## 2. HHP Technology: Principles, Fundamentals and Processing

The industrial HHP treatment process is generally carried out by placing the food to be treated in a hermetically sealed and flexible container and then introducing it into the pressure chamber. Once the processing conditions of pressure level (100–600 MPa) and holding time have been established, the pressure is built up by means of a pump and pressure intensifier and then transmitted to the food via a liquid transfer medium, usually water, that can be recycled after processing. Although it is considered a non-thermal treatment, adiabatic heating must be taken into account [38], which is approximately 3 °C per 100 MPa for water [39]. In addition, combined pressure and temperature treatments can be carried out using temperature control devices and insulated vessels [40,41,42].

The principles on which this technology is based are the isostatic principle, which assumes that the pressure is applied uniformly, instantaneously and homogeneously to the food, and Le Chatelier’s principle, which refers to the application of pressure with an effect on volume leads to a change in the equilibrium of the system [29]. As a consequence of the HHP treatment, the pressurised material may undergo phase transitions, changes in molecular configuration and chemical reactions [43]. On this basis, and depending on the processing conditions, food biomolecules are affected. The impact of pressure on proteins can cause unfolding, partial denaturation or changes in the electronic configuration of some amino acid side chains [44]. In turn, HHP treatment on starch under certain conditions of pressure level, starch:water ratio and holding time can affect non-covalent interactions, leading to changes at the supramolecular level and, hence, on their techno-functional properties [45]. The following section describes the effect of HHP treatments on starch and protein, the two main biopolymers present in flours and cereal derivatives, in more detail.

## 3. Impact of HHP Treatments on the Main Biopolymers of Starchy Raw Materials

### 3.1. Effect of HHP Treatments on Starch

The emerging interest in the physical modification of native starches is based on the need to improve their functionality in baked goods with reduced chemical additive content [46,47]. Non-thermal technologies, such as HHP, can meet this purpose for their ability to disrupt the granule crystallinity in the presence of water, enabling new functionalities together with the generation of new label-friendly ingredients [48]. Depending on the botanical origin of the starch and its amylose content, the presence of water and the HHP processing conditions (pressure, holding time and temperature), the starch modification effect or the degree of gelatinisation achieved may be variable [49]. In order to understand the mechanisms underlying the impact of HHP on this biomolecule, a number of recent investigations have focused on this area.

At the atomic level, an investigation made using molecular dynamics simulation explored the changes induced in the starch molecule conformation at different levels of applied pressure [50]. In that study, an increase in molecular stability was found as the fluctuation range (root mean square fluctuation) of the molecules decreased due to pressure. The authors also observed changes in the conformation of amylopectin and amylose with increasing pressure in terms of a reduction in the distance between the amylopectin chains and the two double amylose chains. They explained that this effect could be related to deformations (holes and cavities) on the starch granule surface promoted by the HHP treatment. With increasing pressure, they also reported antagonistic changes of non-covalent bonding forces at the level of supramolecular structure, resulting in the alteration of the native crystalline starch structure. This could be associated with changes in X-ray diffraction patterns [46] as well as with the disappearance of birefringence patterns [29]. The influence of HHP treatment on the ordered state of crystallinity with different amylose/amylopectin ratios in maize starches has been investigated [51]. A significant reduction in the SAXS (small-angle X-ray scattering) peak area of the waxy and normal maize starches compared to those with high amylose content (B-type) was observed. This higher resistance to compression of B-type starches has been attributed to the shorter amylose linkages, which leave less space for compression through the lamellar structure and limit the flexibility to absorb internal stresses. The more open helices arrangement of B-type starches allows larger amounts of water molecules accommodation (36 instead of 8 for A-type), resulting in stronger hydrogen bond networks to stabilise the helix structure against pressure forces. On the other hand, A-type starches have scattered branching points within the crystalline region, establishing “weak points” in the granular structure and making it more vulnerable. Therefore, the A-type structure typically presented on cereals (such as rice, corn and wheat) and pseudocereals (buckwheat) is more sensitive to being gelatinised by HHP treatment [52].

The process of water molecules entering and binding to starch molecules, together with the weakening of starch intramolecular hydrogen bonds, is driven by compressive forces once they exceed a certain threshold, allowing the existing structure to be disrupted and starch gelatinisation to begin. The effects of pressure on starch at a micron-size granule level have been extensively studied and are generally represented by a wide range of changes in the morphological and functional properties of starch granules, particularly in their swelling and solubilisation properties [45,53]. Similar to heat-driven gelatinisation, in which hydration, swelling of the amorphous region and loss of birefringence processes occur, in the pressure-driven gelatinisation process, the crystalline regions are prevented from melting because amylose helps to stabilise amylopectin, thus interrupting starch gelatinisation of the crystalline region and maintaining the granular conformation [54]. However, at high enough pressure, total gelatinisation can occur [55], even for B-type starches [51]. The extent of gelatinisation can be modulated by processing conditions (starch:water ratio, pressure level, holding time, temperature) [51], allowing intermediate levels of crystalline degradation or partial gelatinisation to be obtained, with different changes in starch functionality.

### 3.2. Effect of HHP Treatments on Protein

Studies on the effects of HHP treatments on biomolecules started in the 1960s and were focused on the impact on proteins, nucleoproteins and membranes of pressure-sensitive microorganisms [56]. The stability of biosystems under high pressure could be predicted by Le Chatelier’s principle, as the application of pressure will shift the biosystem to a new equilibrium state occupying a smaller volume through molecular interactions [57]. The structural thermodynamic equilibrium of proteins depends mainly on three types of interactions: ionic, hydrophobic and hydrogen bonding. Ion pairs are strongly influenced by the pressure in an aqueous solution, resulting in the arrangement of water molecules in their vicinity due to the electrostriction compression effect. Hydrophobic groups can be similarly affected when the pressure level causes protein unfolding, exposing hydrophobic residues and facilitating interactions between them through van der Waals forces. In addition, the application of HHP has been related to the formation of hydrogen bonds with small changes in the activation volume [56].

The effect of pressure on protein unfolding is different from that driven by temperature. The thermal process may completely and irreversibly unfold the protein, breaking covalent bonds and displacing non-polar hydrocarbons towards the solvent medium. On the other hand, pressure rarely alters covalent bonds but mainly affects the tertiary and quaternary structures of proteins. The pressure-unfolding mechanism begins when pressure forces induce water molecules to enter the interior of the protein, destabilising non-polar groups. The pressure sensitivity of proteins, therefore, depends on the conformational flexibility of their structure, which is maintained despite the loss of some non-polar domains due to the inclusion of water molecules [54]. It has been reported that at pressures above 200 MPa, changes occur in the protein structure of globulins, leading to aggregation as a result of protein–protein interactions. In contrast, below 200 MPa, only some tertiary and quaternary conformational changes occur, as these pressure levels affect weak bonds, such as van der Waals’ forces, hydrophobic interactions and electrostatic and hydrogen bonds. Depending on the protein, pressure application above 300 or 400 MPa generally leads to irreversible denaturation. Therefore, according to the most abundant protein fraction, structural pressure-induced changes could have a major impact on protein functionality, with changes in solubility and hydration behaviour, interfacial activity and rheological properties [23].

## 4. Impact of HHP Treatments on Techno-Functional Properties of Starch and Protein Biopolymers

### 4.1. Effect of HHP Treatments on Techno-Functional Properties of Starch

Starch is a polymeric carbohydrate consisting of numerous glucose units linked by glycosidic bonds and is the most abundant and important carbohydrate in flours. Starchy foods are the primary source of carbohydrates for most people, and starch provides basic functionality for the development of common bakery products. However, native starches do not always offer the functionalities currently required for the food product development industry, especially in complex formulations where the use of chemical additives is reduced, or even clean-label food products are desired [48]. In this context, there is growing interest in enhancing the functionality of starches through physical modification. Improvements in swelling, solubility or gelatinisation are the main areas of interest in starch modification. HHP technology has the ability to modify the structure of the starch molecule, facilitating water entry into crystalline regions due to the effect of pressure weakening the double helix [24]. Pressure increases the water diffusion into the starch amorphous region, leading to crystal disruptions. However, pressure gelatinisation depends on extrinsic conditions such as starch type and hydration [55] and intrinsic processing conditions such as pressure level, temperature and holding time [48]. This pressure gelatinisation differs from heat-induced gelatinisation, in which the amylose and amylopectin molecules and residual granules are solubilised to form a starch paste [58]. In contrast, in pressure-induced gelatinisation, the starch granules are deformed but retain their granular shape [59]. Table 1 shows the available results on the changes induced by HHP treatment on the techno-functional properties of plant starches according to the treatment conditions applied.

The degree of gelatinisation achieved by pressure treatment correlates with water-binding capacity (WBC), as reported by Rumpold and Knorr [60], who observed that wheat, tapioca and starch suspensions (5%) increased their WBC with increasing pressure. When comparing fully gelatinised samples, the highest WBC was observed for tapioca starch, followed by potato, but at 450 MPa, the wheat starch sample showed the highest WBC because, unlike the other treated samples at that pressure level, it was completely gelatinised [60]. Increased water retention capacity with the pressure has also been reported for other starch sources, such as corn [34] and quinoa [61]. The latter authors related this increase to the observed increase in damaged starch. They also stated that pressure-damaged starch was more easily swollen. However, the hydration behaviour of HHP-modified starch granules could be different depending on the test temperature. Li et al. [55] reported higher swelling power and water solubility at 50–60 °C of rice starch treated with HHP (600 MPa for 30 min at room temperature) compared to native rice starch. However, at test temperatures above 70 °C, the HHP-treated rice starch showed an opposite effect with a decrease in swelling and solubility [55]. This behaviour could be caused by an aggregation of amylose molecules due to the effect of pressure, which favoured the promotion of lipid–starch associations at 50–60 °C, leading to an increase in water retention capacity and solubility. However, at higher temperatures, the ordered state of the rearranged amylose molecules may have prevented them from melting, limiting amylopectin swelling and amylose solubilisation. This behaviour was consistent with the findings for HHP-treated common buckwheat starch [62]. However, this trend was not followed for quinoa and pea starch, where higher solubility was found at higher temperatures [46,61].

Variations in the pasting profiles of HHP-treated starches have also been reported in the literature, depending on the starch source and the treatment conditions. B-type diffraction pattern starches, such as those found in potatoes, were more resistant to pressure treatment [63]. Conversely, starches with A-type diffraction patterns, such as those of cereals, were more sensitive to pressure and showed significant changes in the pasting profile [55]. Li et al. [55] reported different pasting profiles of HHP-treated rice starch depending on the pressure level. At pressures below 480 MPa, the peak, trough and final viscosities were higher than those observed for native rice starch. These authors associated the increase in these viscosities with the increase in the swelling power of these granules, which had a fragmented crystalline structure. However, a significant drop in maximum, minimum and final viscosity was also observed for starch samples treated with HHP at 600 MPa [55]. They explained that at this level of pressure, the amylose and lipid developed a helical complex that intertwined with the amylopectin molecules, limiting their ability to swell and preventing them from melting, improving their paste stability [55]. A similar pasting behaviour at 600 MPa was also observed for pea starch [46], corn and quinoa starch [64], as well as for buckwheat starch [62].

**Table 1:** Technological changes in starch promoted by high-hydrostatic-pressure treatments.

Botanical Starch Source	HHP Treatment Conditions	Effect on Techno-Functional Properties	Reference
**Cereals**			
Wheat	P: 0.1–500 MPa Time: 15 min T: 25–66 °C S/W: 5% (*w*/*w*)	Increased water-binding capacity.	[60]
Barley	P: 400–550 MPa Time 0–75 min T: 30 °C S/W: 10% and 25% (*w*/*w*)	Increased gel consistency with the pressure and holding time (10%). G′ increased with the pressure (25%). G′ increased with longer holding times (25% *w*/*w*, P = 400–450 MPa).	[65]
Corn	P: 400–600 MPa Time: 5–10 min T: 20–40 °C S/W: 40% (*w*/*w*)	Increased water and sodium carbonate retention capacity. Increased sucrose retention capacity. Decreased breakdown viscosity (P = 600 MPa). Increased setback viscosity (P= 600 MPa, 40 °C, time: 10 min).	[34]
Rice	P: 120–600 MPa Time: 30 min T: room temperature S/W: 20% (*w*/*w*)	Increased swelling power and solubility (P = 600 MPa, 50–60 °C). Decreased swelling power and solubility (P = 600 MPa, 70–90 °C). Increased peak, trough and final viscosities (P = 120–480 MPa). Increased pasting temperature and decreased peak, breakdown and setback viscosities (P = 600 MPa).	[55]
Sorghum	P: 300–600 MPa Time: 10 min T: 20 °C S/W: 25% (*w*/*w*)	Increased complex viscosity values at the beginning of gelatinisation. Decreased breakdown viscosity (P > 300 MPa).	[66]
**Tubers**			
Potato	P: 400–600 MPa Time10 min T: 21 °C S/W: 1:3 (*w*/*w*)	Increased peak and breakdown viscosities (P = 400 MPa). Increased final viscosity and peak time (retrograded samples for 7 days at 4 °C).	[63]
**Roots**			
Tapioca	P: 400–600 MPa Time: 15–30 min T: 22–25 °C S/W: 1:3, 1:4 (*w*/*w*)	Increased G′ values with increasing the pressure level and concentration. Increased gel firmness with increasing the holding time. Increased mechanical strength with increasing the concentration.	[67]
**Pseudocereals**			
Quinoa	P: 300–600 MPa Time: 15 min T: 26 °C S/W: 1:3; 1:4 (*w*/*w*)	Increased water-holding capacity (P = 600 MPa). Increased water solubility index (from 1.99 to 3.45%). Increased G′ with increasing the pressure and starch concentration.	[61]
	P: 100–600 MPa Time: 5 min T: room temperature S/W: 10% (*w*/*v*)	Decreased water solubility index at 55–95 °C (P ≥ 500 MPa). Decreased swelling power at 75–85 °C (P ≥ 500 MPa). Increased swelling power at 55–65 °C (P ≥ 500 MPa). Decreased consistency coefficient (K) (P ≥ 500 MPa). Increased G′ and decreased G″ (P ≥ 500 MPa). Decreased pasting temperature (P = 600 MPa). Decreased peak viscosity (P ≥ 500 MPa). Increased peak viscosity (P = 500 MPa).	[64]
Buckwheat	P: 120–600 MPa Time: 20 min T: room temperature S/W: 20% (*w*/*v*)	Increased swelling power and solubility at 50–60 °C (P ≥ 360 MPa). Decreased swelling power at 70–90 °C (P ≥ 120 MPa). Decreased hardness, adhesiveness, gumminess and chewiness of starch gels. Increased pasting temperature and peak time. Decreased peak, breakdown and setback viscosities.	[62]
**Legumes**			
Pea	P: 150–600 MPa, Time: 25 min T: 30 °C S/W: 15% (*w*/*w*)	Increased water absorption and solubility index and swelling power. Increased peak, breakdown and setback viscosities (P = 150–450 MPa). Decreased peak, breakdown and setback viscosities (P = 600 MPa).	[46]

P: pressure; T: temperature; S/W: starch-to-water ratio.

### 4.2. Effect of HHP Treatments on Techno-Functional Properties of Proteins

As was previously explained, pressure forces can alter the protein functional groups by affecting their quaternary, tertiary and even secondary structure [68]. These modifications can lead to changes in water retention capacity, emulsifying and foaming properties and viscoelastic behaviour, which could be used to improve breadmaking performance in a similar way to chemical additives. Table 2 shows the findings reported in the literature on the techno-functional changes in proteins promoted by HHP treatments.

Different studies have reported an improvement in the hydration properties of HHP-treated proteins [68,69,70,71]. An increase in the water-holding capacity of pine nut protein fractions [70] and kidney bean protein isolates [68], as well as in the water absorption capacity of rice bran proteins [69] with increasing pressure levels, has been observed, which has been attributed to protein unfolding. The loss of structure favoured the increase of exposed functional hydrophilic groups, providing more water-binding sites. In addition, this unfolding of proteins may also expose inaccessible hydrophobic groups from the protein core. This structural change may also improve the oil absorption capacity of proteins, as has been observed in HHP-treated rice bran proteins [69] and pine nut proteins [70].

Protein solubility is one of the most important properties from a techno-functional point of view and plays an important role in other properties such as emulsion, foaming and gelling capacity [72]. It has been reported that pressure, together with enzymatic hydrolysis, can reduce the size of peptides by breaking peptide bonds and thus increase solubility [28]. Zhu et al. [69] observed significant changes in the solubility of isolated rice bran proteins associated with the pressure level applied, with a significant increase in solubility observed in samples pressurised between 100 and 200 MPa and a decrease at pressures above 200 MPa. It was suggested that the increasing result observed was due to the partial opening of protein structures at low pressure levels [69]. Similarly, an increase in the solubility of pine nut protein fractions was also observed with HHP treatment, particularly at 200 MPa. This effect was attributed to pressure-induced changes in the spatial structure of the proteins [70]. However, higher pressures resulted in the formation of protein structures that hindered solubility [69]. Qin et al. [73] and Li et al. [71] attributed a decrease in solubility in walnut and soybean protein isolates for the generation of agglomerates produced at pressures above 400 MPa and 300 MPa, respectively.

Contradictory results have been reported on the effect of protein unfolding in relation to the foaming capacity of proteins after HHP treatment. Zhu et al. [69] observed improvements in the foaming capacity of rice bran protein treated with HHP. The authors related this effect to an increase in surface hydrophobicity caused by the unfolding of protein. However, contrary results were observed for the foaming capacity after HHP treatments in kidney bean protein isolate [68], pea protein isolate [74] and soybean protein isolate (P > 300 MPa) [71]. Chao et al. [74] suggested that the decrease in foaming capacity is induced by pressure-mediated protein unfolding, which led to a reduction in protein flexibility and an aggregation between them, hindering their ability to encapsulate air bubbles.

Different results have also been reported for the emulsifying capacity of pressure-treated proteins, depending on the level of pressure applied. While at moderate pressures (100 MPa for rice bran protein and 200–400 MPa for bean protein isolate), the emulsion capacity increased; at higher pressures, there was no increase, or there was even a decrease in this property reported [68,69]. Similar results were obtained with walnut [73] and soy protein isolates [71], as a decrease in the emulsion activity index was observed with increasing pressure levels. These authors attributed the improvement in emulsifying at moderate pressure levels to an increase in the degree of protein unfolding, which could lead to a larger surface area for the oil/water interface. The observed decreases in emulsion stability were attributed to a decrease in the molecular flexibility of the proteins due to the formation of aggregates induced by disulphide bonds [28,71].

Regarding rheological properties, viscoelastic moduli of the HHP-treated wheat proteins showed different results. After HHP treatment at 500 MPa (60 °C), glutenin showed a two-fold increase in the elastic modulus (G′), whereas the viscoelastic moduli of gliadin decreased by approximately 50% [75]. These results were attributed to the higher pressure sensitivity of glutenin compared to gliadin due to its higher thiol group content, which could increase disulphide cross-linking. This is in line with the findings of Cao et al. [70] for pine nut proteins, where a higher viscoelastic modulus was observed with a pressure treatment of 400 MPa. These authors also associated the pressure-induced cross-linking with improvements in protein gel strength. In the study of Kieffer et al. [75], the pressure treatment at 400 MPa (40 °C) led to an increase in the resistance to extension and a decrease in extensibility of gluten at longer holding times.

**Table 2:** Technological changes in proteins promoted by high-hydrostatic-pressure treatments.

Protein Source	HHP Treatment Conditions	Effect on Techno-Functional Properties	Reference
**Cereals**			
Gluten, gliadin and glutenin	P: 0.1–800 MPa Time: 5–30 min T: 30–80 °C P/W: Hydrated in excess of water	Increased gluten resistance to extension and decreased extensibility (P = 400 MPa, 40 °C). Decreased gluten extensibility, cohesiveness and strength (P ≥ 600 MPa). Decreased G′ and G″ (gliadin) (P = 500 MPa). Increased G′ and G″ (glutenin) (P = 500 MPa).	[75]
Rice bran protein	P: 100–500 MPa Time: 10 min T: 20 °C P/W: 1% with phosphate buffer (50 mM, pH 7) (*w*/*v*)	Increased protein solubility (P ≤ 200 MPa/pH 2–3; pH 6–10). Decreased protein solubility (P > 200 MPa). Increased water absorption capacity (P = 500 MPa). Increased oil absorption capacity (P = 200 MPa). Increased foam capacity (P = 500 MPa). Increased foam stability. Increased emulsifying activity (P = 100 MPa). Increased emulsifying stability (P ≤ 400 MPa). Decreased least gelation concentration (P = 200 MPa).	[69]
**Tubers**			
Potato protein concentrate (PPC) and isolate (PPI)	P: 200–600 MPa Time: 10 min T: 20–40 °C P/W: 1% (*w*/*w*) pH 6/7 with 0.1 M hydrochloric acid/sodium hydroxide	Decreased PPC (P > 400 MPa, 40 °C and pH 7) and PPI solubility (P ≥ 400 MPa, pH 6). No change in the time required for foam formation. Increased foam instability.	[76]
Sweet potato protein	P: 400 MPa Time: 30 min T: 25 °C P/W: 4% with Tris-HCL buffer (50 mmol·L^−1^, pH 7) (*w*/*w*) With or without addition of salts (NaCl, MgCl_2_, CaCl_2_)	Increased G′ with the addition of salts. Increased water-holding capacity (WHC) with NaCl. Decreased WHC with MgCl_2_ and CaCl_2._	[77]
**Legumes**			
Kidney bean protein isolate	P: 200–600 MPa Time: 15 min T: 23 °C P/W: 1:4; 1:5 (*w*/*w*)	Increased water-holding capacity. Decreased foaming capacity (from 76.7 to 42.1%). Increased emulsifying activity and stability. Increased elastic-like rheological behaviour with increasing protein concentration.	[68]
Pea protein isolate (PPI)	P: 200–600 MPa Time: 5 min T: 23 °C P/W: 1%, PPI: phosphate buffer (pH 7) (*w*/*v*)	Decreased oil droplet size (better emulsion quality) (P = 600 MPa, pH 3). Increased emulsion stability (P = 600 MPa, pH 3 and pH 7). Increased foaming capacity.	[74]
Soy protein isolate (SPI)	P: 200–500 MPa Time: 15 min Time: 5–20 min (300 MPa) T: 20 °C P/W: 1% (*w*/*v*) (pH 6.8)	Increased solubility, water-holding capacity, emulsion activity index and foam capacity (P = 200–300 MPa and 5–15 min). Decreased solubility, water-holding capacity, emulsion activity index and foam capacity (P > 300 MPa and 20 min at P = 300 MPa. Decreased emulsion stability index and foam stability.	[71]
**Nuts**			
Pine nut protein isolate	P: 100–400 MPa Time: 10 min T: 20 °C P/W: 10% (*w*/*v*)	Increased solubility (P = 200 MPa). Increased water- and oil-holding capacity (P = 400 MPa). Increased gel consistency (G*) (P = 100/400 MPa). Increased gel resistance to the strain (P = 400 MPa).	[70]
Walnut protein isolate	P: 300–600 MPa Time: 20 min T: room temperature P/W: 1% with phosphate buffer (0.2 mol·L^−1^, pH 8) (*w*/*v*)	Decreased solubility with increasing pressure. Increased foaming capacity and foaming stability. Increased emulsion activity index (P ≤ 400 MPa). Decreased emulsion activity index (P > 400 MPa). Decreased emulsion stability index.	[73]

P: pressure; T: temperature; P/W: protein-to-water ratio.

## 5. Impact of HHP Treatment in Complex Matrices

As shown in the previous section, depending on the processing conditions, the HHP technology could produce functionalities in starch and proteins that could be of interest for improving baking performance. As a complement to the HHP treatments carried out on these biopolymers, it is also interesting to develop treatments for more complex matrices such as flours, where starch and protein are complemented by other constituents such as fibre [2]. In these systems, the effects of HHP treatments on their functional properties are determined by their complex composition and differ from those achieved by treatments on isolated polymers. Ahmed et al. [78] found that the protein-free rice starch suspension was completely gelatinised at 550 MPa, whereas its rice flour counterpart required 650 MPa for the same holding time. Sharma et al. [79] reported that the presence of protein could decrease the degree of starch gelatinisation. This could be due to the effect of water competition between starch and protein, leaving less water available for starch gelatinisation by HHP [59]. In addition, this effect could be even more prominent if the HHP-modified protein had increased its water-binding capacity [68,69]. Similarly, the presence of fibre has been shown to reduce starch gelatinisation [80]. Therefore, since complex formulations, such as flours, exhibit changes in their functional behaviour due to possible interactions between their components [81,82,83], several studies have been carried out to elucidate the techno-functional response of HHP application in these matrices. Table 3 shows the HHP impact on the techno-functional properties of the resulting flours.

For pressure-treated whole wheat flour and jasmine rice flour, a linear increase in water-holding capacity was found with increasing pressure level and flour-to-water ratio (from 1:1 to 1:4; *w*/*w*) as was shown by Ahmed, Mulla and Arfat and Ahmed, Mulla, Arfat et al., respectively [35,84]. In addition, an increase in the water-holding capacity of non-hydrated wheat flour (14.6% moisture content) was also observed with increasing pressure and holding time [85]. In these reports, changes in hydration behaviour were attributed to alterations in particle size due to HHP treatment, with an increase in the surface area as a reduction in particle size was observed. Ahmed, Mulla and Arfat [35] also suggested that pressure favoured damaged starch granules, thus facilitating their swelling. In other studies in which a pressure treatment was applied to pre-soaked grains of whole grain suspensions of brown rice and buckwheat, an increased water absorption capacity of the resulting flours was also found [37,86]. In those studies, it was suggested that the HHP treatment might have increased the hydrogen bonds between water and starch molecules, thus increasing their water absorption capacity. However, in contrast to the results reported by Gutiérrez et al. [37], Zhu et al. [86] observed a decrease in the swelling power of brown rice flour samples measured at 50 °C. This decrease was attributed to the presence of dispersed fibres partially destroyed by the pressure treatment which inhibited the swelling of the brown rice flour.

Unlike other reports [68,69,74], a decrease in the foaming and emulsifying properties of the resulting buckwheat flours was observed after HHP treatments on whole grains. As a result, it was proposed that the application of pressure promoted changes, leading to a loss of surfactant properties. The authors suggested that it could be related to changes in the distribution patterns of hydrophilic/hydrophobic groups of proteins [37]. It has been reported that HHP may alter the balance of non-covalent bonds, increasing the exposure of functional groups such as disulphide groups. This could lead to the stretching of the protein molecules [70], reducing their flexibility with the cross-linking of disulphide bonds and losing efficacy in emulsion formation, as stated by Cabra et al. [87].

Numerous reports have shown that the impact of HHP treatments led to an overall modification in the pasting viscosity profiles of the flours. A reduction in peak, breakdown and setback viscosities in HHP-treated flours has been reported for wheat [35,88,89], rice [84], waxy rice [34], sorghum [89] and buckwheat [37]. In pressure-treated legume flour, a decrease in pasting temperature has also been reported for green pea and chickpea samples, but it was not always possible to obtain an RVA profile as it depends on the starch content of the sample [58]. Hence, HHP treatments may lead to changes in the starch molecules that would be detected in RVA tests. The extent of these changes would be associated with the mechanisms that facilitate pressure gelatinisation and the HHP treatment conditions. Thus, if a pressure level threshold is not reached, the gelatinisation process will not occur [90]. In addition, the presence of water is also required [59]. Therefore, the higher the pressure and water availability, the higher the degree of gelatinisation that can be achieved [88], as this allows the infiltration of water into the starch molecule, leading to a partial gelatinisation of the inner regions of the starch granule [89]. The degree of gelatinisation achieved could be measured by a decrease in enthalpy. However, in composite matrices, it has been observed that the pressures required in flour are higher than in starch to induce enthalpy changes [88]. Zhu et al. [86] have suggested that the decrease in enthalpy should be attributed to a combination of starch gelatinisation and protein unfolding. The decrease in peak viscosity observed in flour samples could also be a characteristic of the presence of pre-gelatinised starch [88]. Furthermore, it has been suggested that pressure gelatinisation induces changes in the helical structure of the amylose and amylopectin branches, which could lead to a restriction in amylose leaching due to a reinforcement of the granular structure [88]. This might explain the decrease in breakdown viscosity values mentioned above, as the destabilisation effect on the crystallite and subsequent melting in the amorphous region would be reduced [89]. Complementary, Cappa et al. [34] observed that HHP treatments seemed to have a greater effect on those samples with high amylose content, as the higher pasting temperature was attributed to the more compact starch structure generated by the HHP treatment. Similarly, in the study by Gutiérrez et al. [37], the reduction in breakdown viscosity values was attributed to a reinforced crystalline structure due to possible starch protein/fibre entanglement. Consequently, these authors ascribed the reduction in setback viscosity to a decrease in amylose leaching through this reinforced structure, allowing a lower content of free amylose molecules to be further retrograded.

Although a wide variability of results has been observed when analysing the rheological properties of gels made from HHP-treated flours by oscillatory measurements, an overall increase in the elastic modulus (G′) has been observed in wheat [35], oat [91], rice [78], buckwheat and tef [90] and chickpea flours [92,93]. The increase in G′ with pressure has been attributed to the partial gelatinisation effect combined with protein aggregation [35,94]. Some authors have also pointed out the importance of the flour-to-water ratio (F/W) in increasing the mechanical strength of the gel. Ahmed et al. [35] suggested that the higher complex viscosity (η*) values observed at higher concentrations of whole wheat flour were caused by higher molecular interactions and a strengthened structure with increasing pressure. Similar conclusions were reached by Ahmed et al. [78] and Alvarez et al. [92] when they observed the same trend in basmati rice flour and chickpea slurries, respectively. However, Hüttner et al. [91] attributed the increase in G′ observed in the oat dough treated at pressures above 350 MPa mainly to the swelling of the starch granules. On the other hand, a decrease in viscoelasticity was observed in a Thai jasmine rice flour dispersion at 600 MPa with increasing F/W ratio from 1:1 to 1:4. The plasticising effect of the rice flour was attributed to an increased shear-thinning behaviour with increasing pressure [84].

**Table 3:** Technological changes on diverse, complex matrices (flours and grains) promoted by HHP treatments.

HHP-Treated Complex Matrix	HHP Treatment Conditions	Effect on Techno-Functional Properties	References
**Cereals**			
Wheat flour	P: 200–600 MPa Time: 5 min T: 25 °C F/W: 33–56% of moisture content	Decreased pasting profile viscosities (56%).	[88]
	P: 0.1–600 MPa Time: 10 min T: room temperature F/W: 14.6% of moisture content	Increased water retention capacity (from 65.68 to 73.77%).	[85]
Wheat flour (whole)	P: 300–600 MPa Time: 10 min T: 26–38 °C F/W: 1:1; 1:2; 1:3, 1:4 (*w*/*w*)	Increased water-holding capacity. Increased water solubility index. Increased texture hardness and decreased stickiness. Decreased peak, breakdown and final viscosities (1:2, *w*/*w*). Increased G′.	[35]
Wheat/oat/millet and sorghum flours	P: 0.1–500 MPa Time: 10 min T: 20 °C F/W: 1:0.6 and 1:1 (*w*/*w*)	Decreased peak viscosity values (wheat, oat, sorghum). Decreased breakdown and setback viscosities (wheat and sorghum). Increased breakdown and setback viscosities (oat).	[89]
Oat flour	P: 200–500 MPa Time: 10 min T: 20 °C F/W: 1:0.95 (*w*/*w*)	Increased extension of the linear viscoelastic region. Yield stress increased. Decreased loss tangent (P ≥ 350 MPa).	[91]
Rice and waxy rice flours	P: 400–600 MPa Time: 5–10 min T: 20–40 °C F/W: 40% of moisture	Decreased peak viscosity (rice flour). Increased pasting temperature (P = 600 MPa.) Decreased peak viscosity (rice flour). Decreased breakdown viscosity.	[34]
Basmati rice flour	350–650 MPa 7.5–15 min T: 22–26 °C F/W: 1:2, 1:3, 1:5 (*w*/*w*)	Increased G′ (with pressure and holding time). Increased gelatinisation degree.	[78]
Thai jasmine rice flour	P: 300–600 MPa Time: 10 min T: 25 °C F/W: 1:1, 1:3, 1:4 (*w*/*w*)	Increased water absorption capacity and solubility index (with increasing the flour-water ratio and the pressure level). Decreased peak viscosity (F/W = 1:2). Decreased pasting temperature (P ≥ 400 MPa). Decreased trough and breakdown viscosities. Decreased setback viscosity. Increased G′ (F/W = 1:3, *w*/*w*). Increased complex viscosity (η*) values.	[84]
Brown rice grain	Pre-soaking (30 °C, 3 h) P: 200–500 MPa Time: 5–15 min T: room temperature G/W: 1:1.6 (*w*/*v*)	Increased water absorption (from 6.2 to 21.3%). Decreased swelling power (50 °C). Increased swelling power (70 °C). Increased solubility values (70–90 °C).	[86]
Sorghum flour	P: 200–600 MPa Time: 10 min T: 20 °C F/W: 40% (*w*/*w*)	Increased complex viscosity (P > 300MPa). Decreased complex viscosity (P < 300 MPa). Increased loss tangent (P = 200–300 MPa). Decreased loss tangent (600 MPa).	[95]
**Pseudocereals**			
Buckwheat and tef flour	P: 200–600 MPa Time: 10 min T: 20 °C F/W: 40% (*w*/*w*)	Increased pasting temperature (tef batters at 400 MPa). Decreased breakdown and setback. Increased complex modulus and decreased loss tangent (tan δ) (buckwheat). Decreased complex modulus and increased tan δ (tef batters up to 200 MPa). Increased complex modulus and decreased tan δ (tef batters at >200 MPa).	[90]
Unhulled buckwheat grains	Pre-soaking of the grains (40 °C, 4 h) P: 600 MPa Time: 30 min (1 cycle), 15 min (2 cycles) T: room temperature G/W: 1:4; (*w*:*w*)	Increased water absorption capacity (12%) and swelling power (pre-soaking). Decreased emulsifying and foaming capacities and stabilities. Decreased peak (18%), breakdown (93%) and setback (29%) viscosities (with pre-soaking and 1 cycle). Decreased complex modulus (1 cycle and pre-soaking).	[37]
**Legumes**			
Chickpea, green pea and soybean flours *.	P: 200–450 MPa Time: 10 min T: 20 °C F/W: 1:0.6, 1:1 (*w*:*w*)	Decreased breakdown viscosity (chickpea and green pea; F/W = 1:1). Increased peak viscosity and holding strength (chickpea and green pea). Decreased pasting temperature (chickpea and green pea). * The soybean flour samples did not allow a regular RVA profile to be obtained due to the low starch content.	[58]
Chickpea flour	P: 150–600 MPa, Time: 15 min T: 25 °C F/W: 1:2, 1:3, 1:4, 1:5 (*w*:*w*)	Increased G″ (P = 600 MPa). Increased G′ values (1:2 and 1:3, P = 600 MPa). Decreased loss tangent (1:2, P = 600 MPa).	[92]
	P: 200–600 MPa Time: 5–25 min T: 10–50 °C F/W: 1:5 (*w*:*w*)	*For heat-induced gels at 75* °*C:* Increased G′ and G″ at 50 °C, P = 600 MPa, 5 min Increased G′ at 10 °C, P = 200 MPa, 25 min Decreased G′ and G″ for the rest of HHP treatment conditions *For heat-induced gels after storage 1 week (4 °C):* Increased G′ and G″ at 10 °C, P = 200 MPa, 5–15 min; ii. P = 400 MPa, 5–25 min. Increased G′ and G″ at 25 °C, P = 200 MPa, 25 min. Increased G′ and G″ at 50 °C, P = 200–400 MPa, 25 min. Increased G′ at 25 °C, i. P = 200 MPa, 15 min; ii. P = 400 MPa, 5 and 25 min. Increased G′ and G″ at 50 °C, 200 MPa, 15 min Decreased G′ and G″ for the rest of HHP treatment conditions	[93]

P: pressure; T: temperature; F/W: flour-to-water ratio; G/W: grain-to-water ratio.

## 6. Impact of HHP Treatments on Dough Properties and Bread Quality

A number of investigations have been carried out in order to determine the functionality of the HHP-modified ingredients as structure-promoting agents. These include empirical and fundamental rheological tests in doughs that were performed to collect measures such as dough consistency, extensibility, stickiness and/or cohesion, as these properties are closely related to bread quality, particularly in gluten-free formulas [16]. Furthermore, the impact of this physically modified ingredient on leavened bread quality parameters such as specific bread volume, crumb texture or bread staling has also been assessed. The main effects of HHP-treated ingredients on dough rheology and bread quality are summarised in Table 4.

It has been reported that HHP can modify the strength of gluten [75,96]. Therefore, HHP treatments could improve the functionality of wheat flours with poor breadmaking properties [88]. This technology has been proposed to improve bread quality in wheat-based formulations with high-fibre ingredients that are prone to promote detrimental effects [27]. Insoluble fibres lead to physical disruption of the gluten network [2] or create break points where gas can more easily escape during proofing [18].

The importance of applying appropriate HHP conditions to induce higher functionality is of great relevance as numerous studies have found opposite effects. It has been reported that increasing the pressure level and/or holding time increased the viscoelastic modulus of HHP-treated wheat-based cake batter [97]. Similarly, Angioloni and Collar [89] observed significant increases in the storage and loss modulus of doughs containing wheat flours treated with HHP at 350 MPa and above (50% of replacement level). Similar increases were also reported using GF flour (oats, millet and sorghum) treated at 500 MPa for replacement wheat flour between 40 and 60%. In large deformation mechanical tests, wheat-based doughs containing HHP-treated flours resulted in increasing values in hardness and adhesiveness at 150 MPa [96] or at 500 MPa [89]. These authors also reported a loss in dough cohesiveness, an increase in resistance to extension and a decrease in the dough extensibility. Rheological changes could be a consequence of HHP-induced structural changes in starch and protein, such as starch pre-gelatinisation and gluten strengthening through disulphide bond formation [89,98]. Therefore, HHP conditions could lead to an overstructuring effect of the combined action of both structural changes. Kieffer et al. [75] reported higher resistance to extension in gluten samples at high pressures (800 MPa) and temperature conditions (60 °C). This increased resistance could hinder the machinability of the doughs [89]. In this regard, other authors have reported that wheat-based breads containing HHP-modified wheat flour or other cereals such as oats, millet and sorghum showed some detrimental characteristics such as a reduction in specific volume [96], increase in crumb hardness and also a loss in cohesiveness [99].

Angioloni and Collar explored the effect of HHP treatments on legume flours for the possibility of favouring the formation of a protein network through new bonds (e.g., disulphide bonds) despite the generally low methionine, cysteine and tryptophan content of these flours [58,100]. They observed promising structuring effects in HHP treatments (≥350 MPa) on more hydrated legume flour batters (1:1, compared to 1:0.6; *w*/*w*). These effects were related to the formation of structure-promoting disulphide bonds and the formation of urea-insoluble aggregates, in agreement with the observations of Hüttner et al. [91]. The resulting wheat-based breads containing HHP-treated legume batters also showed a decrease in specific volume and a noticeable increase in crumb hardness and staling rate. However, with the addition of hydrocolloid (3% of CMC), not only was the hardening of the breads reduced but also the firming kinetics and overall acceptability were closer to those legume breads used as controls (without HHP treatment), which were highly acceptable [100].

Matsushita et al. [101] reported a significant (*p* < 0.05) increase in the specific volume of strong wheat-based breads obtained by a combined action of HHP treatment (43 MPa) and enzyme supplementation (0.2%) on doughs. Those results were attributed to the action of the enzymatic bakery mixture of α-amylase and hemicellulose, which degraded damaged and gelatinised starch and pentosane, improving gas retention during proofing. In addition, the catalytic activity of the enzymes was improved by HHP treatment at 43 MPa. These breads also had a softer crumb texture compared to a control bread at each storage time measured (1–3 days). Therefore, the combined action of low-pressure treatment and enzymes could be an effective tool to overcome the increase in staling reported in other studies where breads were made with HHP-treated wheat flours [60].

It has been suggested that structure strengthening could be a valid tool to improve the baking performance of gluten-free flour matrices [89]. In these terms, starch pre-gelatinised by HHP treatments has been proposed as a structuring agent [98]. Some promising investigations have been carried out for developing GF breads using HHP-modified flours. Studies have reported improved bread quality properties using GF flours HHP-treated at low pressure levels. Hüttner et al. [102] observed significant increases in bread-specific volume and softer crumb hardness compared to the control and those obtained at higher pressures of breads made with replaced oat flour (10%) with an HHP-treated one at 200 MPa. Similarly, the use of HHP-treated sorghum flour (200 MPa) at the same replacement level had no adverse effect on bread properties [95]. In both investigations, significant increases in the elastic solid behaviour of the doughs were observed with HHP treatments above 350 MPa [102] and 400 [95]. The increase in dough consistency at high pressure levels, which could impair proper bread development, was attributed by Hüttner et al. [102] to the combined action of protein network formation and starch gelatinisation. However, Vallons et al. [95] attributed the strengthening effect mainly to the starch gelatinisation since the rheological test carried out on batters with the addition of NEM (N-ethylmaleimide solution) as a thiol exchange inhibitor had little effect on the rheological properties of the doughs. Conversely, a weaker batter structure was found at 200 MPa. To explain the opposite results found in the batter consistency at 200 MPa of the HHP treatment, both investigations attributed the structural changes occurring in the proteins at this pressure level either to a depolymerisation of the protein [95] or to the weakening of electrostatic and hydrophobic bonds [102]. These authors [102] also suggested that the weakened protein structure did not alter the uniform starch gel network developed during baking. In addition, the modified protein might have improved its foaming properties, as observed by Chao et al. [74], leading to better textural properties of the crumb. Contrary to these studies, the application of HHP treatments at 600 MPa to maize starch or rice flour in a GF bread formulation (with the addition of structuring agents such as HPMC and psyllium) did not impair the bread quality characteristics, as a low specific volume loss (5–7%) was found, and a softer crumb texture was observed. A significant delay in staling was also found [103].

**Table 4:** Impact of HHP-treated ingredients on dough properties and bread quality.

HHP-Treated Ingredient	HHP Treatment Conditions	Effect on Dough Properties	Effect on Bread Quality	References
**Wheat-based formula**				
Wheat starch	P: 600 MPa Time: 15 min T: room temperature S/W: 5% (*w*/*w*)		Slightly increased firmness and decreased elasticity after storage (5 days).	[60]
Wheat flour	P: 50–250 MPa Time: 1–4 min T: room temperature F/W: wheat flour mixed with water up to 500 BU	Increased dough hardness and adhesiveness (P ≥ 100 MPa).	Bigger gas cells with an uneven distribution. Decreased specific bread volume. Increased crumb hardness. Increased moisture content. Reduced luminosity, a* and b* of crust and crumb.	[96]
Wheat flour	P: 200–600 MPa Time: 5 min T: 25 °C F/W: 1:1, 1:2 (*w*/*w*) (33% and 56% of moisture content)	*At 33% of moisture content:* Increased dough strength. Higher development time and stability (P ≥ 400 MPa).		[88]
Wheat flour	P: 0–100 MPa Time: 10 min T: room temperature F/W: wheat flour mixed with water up to 500 BU	*Doughs containing 0.2% bakery enzyme and HHP-treated flour at 43 MPa:* Increased gas retention.	*Doughs containing 0.2% of bakery enzyme and HHP-treated flour at 43 MPa:* Increased specific bread volume. Decreased bread crust luminosity and changed the colourimetric parameters. Improved breadcrumb structure. Reduced hardening kinetics of bread crumbs from dough containing enzymes and HHP-treated flour. Weakened gluten network in breads made from HHP-treated flour without the bakery enzyme.	[101]
Oat, millet, sorghum and wheat flour	P: 200–500 MPa Time: 10 min T: 20 °C F/W: 1:1 (*w*/*w*)	*At 500 MPa:* Increased dough hardness and resistance to extension. Decreased dough cohesiveness (wheat and millet). Decreased extensibility (wheat). Increased viscoelastic moduli (G′ and G″).	*At 350 MPa:* Decreased specific bread volume and crumb cohesiveness. Increased crumb hardness (sorghum flour). Increased overall acceptability (wheat, millet and sorghum flours). Decreased bread staling (wheat and oat flours). Increased bread staling (millet and sorghum flours).	[89,99]
Chickpea, greenpea and soybean flours	P: 200–450 MPa Time: 10 min T: 20 °C F/W: 1:0.6 (*w*/*w*) F/W: 1:1 (*w*/*w*)	*At P ≥ 350 MPa (1:1, w*/*w):* Increased retardation time (chickpea and soybean flours). Decreased instantaneous compliance and increased zero shear viscosity. Reduced stickiness (chickpea and green pea flours).	*At 350 MPa (1:1, w*/*w):* Increased the initial crumb hardness. Decreased specific bread volume. Increased crumb firming kinetics.	[58,100]
**Gluten-free formula**				
Oat flour	P: 200–500 MPa Time: 10 min T: 20 °C F/W: 1:0.95 (*w*/*w*)	*At 10% of replacement level and at P ≥ 350 MPa:* Decreased loss tangent.	*At 10% of replacement level and at 200 MPa:* Increased specific bread volume. *At 40% of replacement level and at 500 MPa:* Decreased specific volume. *At 10, 20 and 40% of replacement level and at 200 MPa:* Decreased crumb hardness at day 5.	[102]
Corn starch, rice flour	P: 600 MPa Time: 5 min T: 40 °C F/W: 1:0.5 (*w*/*w*)		Lower crumb water activity. Decreased specific bread volume. Changes in a* Decreased breadcrumb hardness. Increased moisture retention. Decreased crumb hardness at 24 and 72 h.	[103]
Sorghum flour	P: 200–600 MPa Time: 10 min T: 20 °C F/W: (40% *w*/*w*)		*At 10% and at 600 MPa*: Decreased specific bread volume. *At 2% and 600 MPa:* Decreased crumb hardness after 72 h.	[95]

P: pressure; T: temperature; S-F/W: starch/flour-to-ater ratio.

## 7. High Hydrostatic Pressure (HHP) as a Strategy to Enhance the Nutritional Value of Food Matrices

The use of staple foods as vehicles for dietary micronutrient fortification is widely used as a public health strategy to meet some nutritional needs of the population. Examples include the fortification of white rice, wheat and maize flours. In some countries, fortification of white rice flour with minerals and vitamins is mandatory and is associated with nutritional deficiencies in the population, as the micronutrient-rich bran layer is discarded during rice processing and milling [104]. Some populations, such as those with celiac disease, often show deficiencies in micronutrients and bioactive compounds due to their gluten-free diet, so new approaches are being developed that focus on fortifying gluten-free bakery products [8]. Different methods can be applied to obtain a fortified product, from spray drying, coatings or even direct mixing formulas. Innovative food processing techniques involving minimal or no thermal treatment have gained interest as alternatives for their benefits in the preservation of sensory characteristics and thermolabile bioactive compounds. As a non-thermal pasteurisation technology, HHP is considered to be a processing technique with minimal loss of nutritional and sensory properties [30] and can therefore be considered a suitable technology for micronutrient fortification. Pressure-mediated inward diffusion of nutrients from an enriched medium is the most direct way to enrich the target food, a process known as high-pressure impregnation (HPI) [59]. In this process, nutrients are incorporated into the food product by pressure forces that may impart micro-fractures or affect the permeability of the food material surface, which would facilitate a process of mass transfer by osmotic pressure. Based on this mechanism of action, the concentration of the nutrient in the medium is an important factor to benefit from osmotic phenomena, as well as the state of the food matrix, with a porous or permeable one being highly desirable to increase the diffusion rate [105]. HPI treatments have been used effectively to promote quercetin enrichment in frozen–thawed cranberries [106], calcium in mango cubes [107] and baby carrots [108], curcuminoids in pineapple slices [109] or anthocyanins in apple slices [110]. Since pressure forces can damage cell structures, HHP treatments in stiffer food matrices, such as cereal grains, can also affect their constituent tissues, reducing their natural resistance to mass transfer [111]. In the study of Balakrishna et al. [112], an HPI treatment (600 MPa, 50/70 °C, 5–20 min) was carried out to fortify white rice with thiamine, calcium and zinc. They observed significant increases in the concentration of these nutrients, particularly with increasing temperature and holding time.

The effect of pressure-mediated cell wall damage can also be exploited to improve the bioactive profile of the pressurised food product. Pressures of 30 MPa have been reported to be sufficient to promote cell structural damage in germinated brown rice. Therefore, enzymatic hydrolysis could therefore be accelerated in the denatured substrate, resulting in increased biosynthesis of compounds such as antioxidants (γ-Oryzanol), tricin 40-O-(threo-b-guaiacylglyceryl) ether (TTGE), arabinoxylans, γ-aminobutyric acid (GABA) and vitamins such as E and B [113]. Although it has been shown that enzyme activity can be inhibited at pressures above 100 MPa [113], an increase in antioxidant capacity has been observed in germinated brown rice treated with HHP at 100–500 MPa [114]. These authors attributed this effect to the release of antioxidant compounds bound to cell walls and organelles due to the turbulence and shear effects promoted by the HHP treatment. Similarly, other authors have also reported increases in antioxidant capacity after HHP treatments in alternative food products such as *Prosopis chilensis* seeds [115], sweet potato flour [116] and buckwheat flour [117]. Recent research has also shown positive results with the HHP treatment of whole seeds as an effective and simple method of fortifying flour from the natural compounds found in the outer layers of the seeds. Gutiérrez et al. [37] observed an increase in the phenolic content of buckwheat flour after HHP treatments of whole grains. Furthermore, Balakrishna and Farid [118] showed an increase in the thiamine content of white rice flour after HHP treatment (450/600 MPa, 15/30 min, 50/70 °C) of paddy rice. These authors attributed this improvement to pressure-induced inward diffusion of thiamine naturally present in the outer parts of the seed (natural coat), similar to the infusion provided by the pressure-driven mass transport phenomenon of the HPI treatment. The results of this study concluded that treating whole rice grains with HHP could be an interesting alternative to parboiling for industrial applications.

Few studies are available on the use of HHP-treated flours for the nutritional enrichment of bread in terms of bioactive properties. Positive results in this respect were obtained by Angioloni and Collar [99], who observed a significantly (*p* < 0.05) higher antiradical activity in breads made with HHP-treated wheat, oat, millet and sorghum flours (350 MPa, 10 min, 20 °C) than in those made with native flours.

## 8. Conclusions and Future Directions

The application of non-thermal alternative technologies is a promising area of research to provide a solution for the development of nutrient-rich products. This review presents the effects of HHP technology on plant-based ingredients that can be incorporated into bread formulations. Based on the summarized results, a range of physically modified ingredients (starches, flours or even grains) could be developed by modulating the HHP processing conditions, potentially having a positive impact on the concentration of bioactive compounds. It has been shown that changes in the functionality of these modified ingredients are associated with changes in the main macromolecules of the flours, such as starch and proteins, as HHP causes gelatinization of starch and unfolding of proteins. Establishing appropriate HHP processing conditions is essential to obtain modified ingredients suitable for bread production. With gluten-containing matrices such as wheat flour, it has been observed that the application of high pressure can have the undesirable effects of excessive gluten strength, increased stiffness and resistance to elongation, as well as reduced cohesion. However, significant improvements have been observed with HHP under mild conditions as well as in combination with other treatments. This could be of industrial significance as current industrial HHP devices can operate in this range of processing conditions. On the other hand, promising results have been reported for ingredients used in GF formulations. Depending on the processing conditions, HHP could modify the pasting and rheological behaviour of the GF flour, increasing the thermal stability and elastic properties of the batters which could improve dough expansion and gas retention during proofing, thus enhancing the sensory properties of the nutrient-rich GF bread. Although the fundamentals of the molecular changes caused by HHP treatment have been extensively studied, a broad scope of research is required to fully understand the pressure-induced techno-functional changes in complex matrices to produce customized value-added ingredients for bakery products.

## Data Availability

Not applicable.
